# Supplementary material for: Decreased incidence, virus transmission capacity, and severity of COVID-19 at altitude on the American continent
Source: PLoS One. 2021 Mar 29;16(3):e0237294. doi: 10.1371/journal.pone.0237294 (PMC8006995; doi:10.1371/journal.pone.0237294)
Supplement: S1 Table — (PDF) [file pone.0237294.s001.pdf]

S1 Table. Capitals of American countries located above 1,000 m above sea level.

City	Country	Population	Population density (inhabitants/Km^2)	Average altitude (masl)
La Paz	Bolivia	1,864,191	378	3,600
Quito	Ecuador	1,100,000	475.51	2,850
Bogota	Colombia	6,422,198	24,643	2,625
Mexico City	Mexico	22,000,000	5,966	2,240
Guatemala City	Guatemala	1,700,000	159	1,529
San Jose	Costa Rica	2,723,000	7,548	1,146
Brasilia	Brazil	1,650,000	423.28	1,079
