# Supplementary material for: Decreased incidence, virus transmission capacity, and severity of COVID-19 at altitude on the American continent
Source: PLoS One. 2021 Mar 29;16(3):e0237294. doi: 10.1371/journal.pone.0237294 (PMC8006995; doi:10.1371/journal.pone.0237294)
Supplement: S2 Table — (PDF) [file pone.0237294.s002.pdf]

S2 Table. **Sources of epidemiological data**

Country	Source	URL
Argentina	Ministry of Health	https://www.argentina.gob.ar/coronavirus/informe-diario
Belize	World Health Organization	https://who.maps.arcgis.com/apps/webappviewer/index.html
Bolivia	National Health Information System	https://snis.minsalud.gob.bo/
Brazil	Ministry of Health	https://especiais.g1.globo.com/bemestar/coronavirus/mapa-coronavirus/#/
Canada	University of Toronto	https://github.com/ishaberry/Covid19Canada/blob/master/cases.csv
Chile	Ministry of Health	https://www.minsal.cl/nuevo-coronavirus-2019-ncov/informe-epidemiologico-covid-19/
Colombia	Ministry of Technologies of information and Communication	https://www.datos.gov.co/en/d/gt2j-8ykr/visualization#
Cuba	Pan-American Health Organization	https://who.maps.arcgis.com/apps/webappviewer/index.html?id=2203b04c3a5f486685a15482a0d97a87
Costa Rica	Ministry of Public Health	https://www.salud.gob.ec/el-ministerio-de-salud-publica-del-ecuador-msp-informa-situacion-coronavirus/
Dominican Republic	General Epidemiology Department	https://www.msp.gob.do/web/?page_id=6948
Ecuador	Ministry of Public Health	https://www.salud.gob.ec/actualizacion-de-casos-de-coronavirus-en-ecuador/
El Salvador	Official COVID-19 platform of El Salvador government	https://covid19.gob.sv/
French Guyana	University Johns Hopkins	https://github.com/CSSEGISandData/COVID-19/blob/master/csse_covid_19_data/csse_covid_19_time_series/time_series_covid19_confirmed_US.csv
Haiti	Ministry of Public Health and Population	https://www.mspp.gouv.ht/
Honduras	Office for Presidential communication and strategy	https://covid19honduras.org/
Mexico	National Direction of Epidemiology	https://covid19.sinave.gob.mx/

Country	Source	URL
Panama	Ministry of Health	http://minsa.gob.pa/coronavirus-covid19
Paraguay	Ministry of Public Health and Social Welfare	https://www.mspbs.gov.py/reportes-covid19.html
Peru	Ministry of Public Health	https://covid19.minsa.gob.pe/sala_situacional.asp
Puerto Rico	Health Department of Puerto Rico	https://medicinaysaludpublica.com/covid-19/
Uruguay	National Urgencies System	https://www.gub.uy/sistema-nacional-emergencias/pagina-embebida/visualizador-casos-coronavirus-covid-19-uruguay
United States	University Johns Hopkins	https://github.com/CSSEGISandData/COVID-19/blob/master/csse_covid_19_data/csse_covid_19_time_series/time_series_covid19_confirmed_US.csv
Venezuela	Official Venezuelan government COVID-19 platform	https://covid19.patria.org.ve/estadisticas-venezuela/
