# Supplementary material for: Decreased incidence, virus transmission capacity, and severity of COVID-19 at altitude on the American continent
Source: PLoS One. 2021 Mar 29;16(3):e0237294. doi: 10.1371/journal.pone.0237294 (PMC8006995; doi:10.1371/journal.pone.0237294)
Supplement: S1 Appendix — (PDF) [file pone.0237294.s003.pdf]

## S1 Appendix. Equations and parameters used in the SEIR model.

Total population: $N = S(t) + E(t) + I(t) + R(t)$

$$\frac{dS(t)}{dt} = -\beta S(t)I(t)$$

$$\frac{dE(t)}{dt} = \beta S(t)I(t) - \epsilon E(t)$$

$$\frac{dI(t)}{dt} = \epsilon E(t) - \gamma I(t)$$

$$\frac{dR(t)}{dt} = \gamma I(t)$$

$$\beta = interaction\ frequency\ among\ individuals * probability\ of\ transmission\ of\ the\ disease$$

$$\epsilon = \frac{1}{incubation\ period}$$

$$\gamma = \frac{1}{infectious\ period}$$

Where:

*S(t)*: Fraction of population that is susceptible.
*E(t)*: Fraction of population that is exposed to infection.
*I(t)*: Fraction of the population that is infected.
*R(t)*: Fraction of population that has recovered or died.
$\beta$: Contact rate
$\epsilon$: Rate at which exposed individuals become infected.
$\gamma$: Recovery rate.

| Parameter | Value | Source |
|---|---|---|
| Infectious period | 7.5 days | Comité Operativo de Emergencias UMSA. Situación y proyecciones de COVID-19 en Bolivia: 3er reporte. La Paz - Bolivia: Universidad Mayor de San Andrés, May, 2020. Report No.: 3. |
| Incubation period | 6 days | |
| Interaction frequency | 8.1 | Birbuet JC, López R. Dinámica de expansión del COVID-19 en Bolivia durante las primeras 6 semanas. 2020. |
| Initial number of infected people | 30 | |
