# Supplementary material for: Decreased incidence, virus transmission capacity, and severity of COVID-19 at altitude on the American continent
Source: PLoS One. 2021 Mar 29;16(3):e0237294. doi: 10.1371/journal.pone.0237294 (PMC8006995; doi:10.1371/journal.pone.0237294)
Supplement: S1 Fig — Epidemiological data were retrieved on May 23. Data on population density were extracted from the dataset created by CIESIN [22] or the corresponding country’s national statistics institute on May 23. Data were normalized by the population density of the same location, adjusted by calculating the natural logarithm (ln) of each value, and summed in intervals of 100 m of elevation. Raw, normalized, and adjusted data are available at https://doi.org/10.6084/m9.figshare.12685478. (PDF) [file pone.0237294.s004.pdf]

S1 Fig. **Effect of altitude in the incidence of COVID-19 per countries**

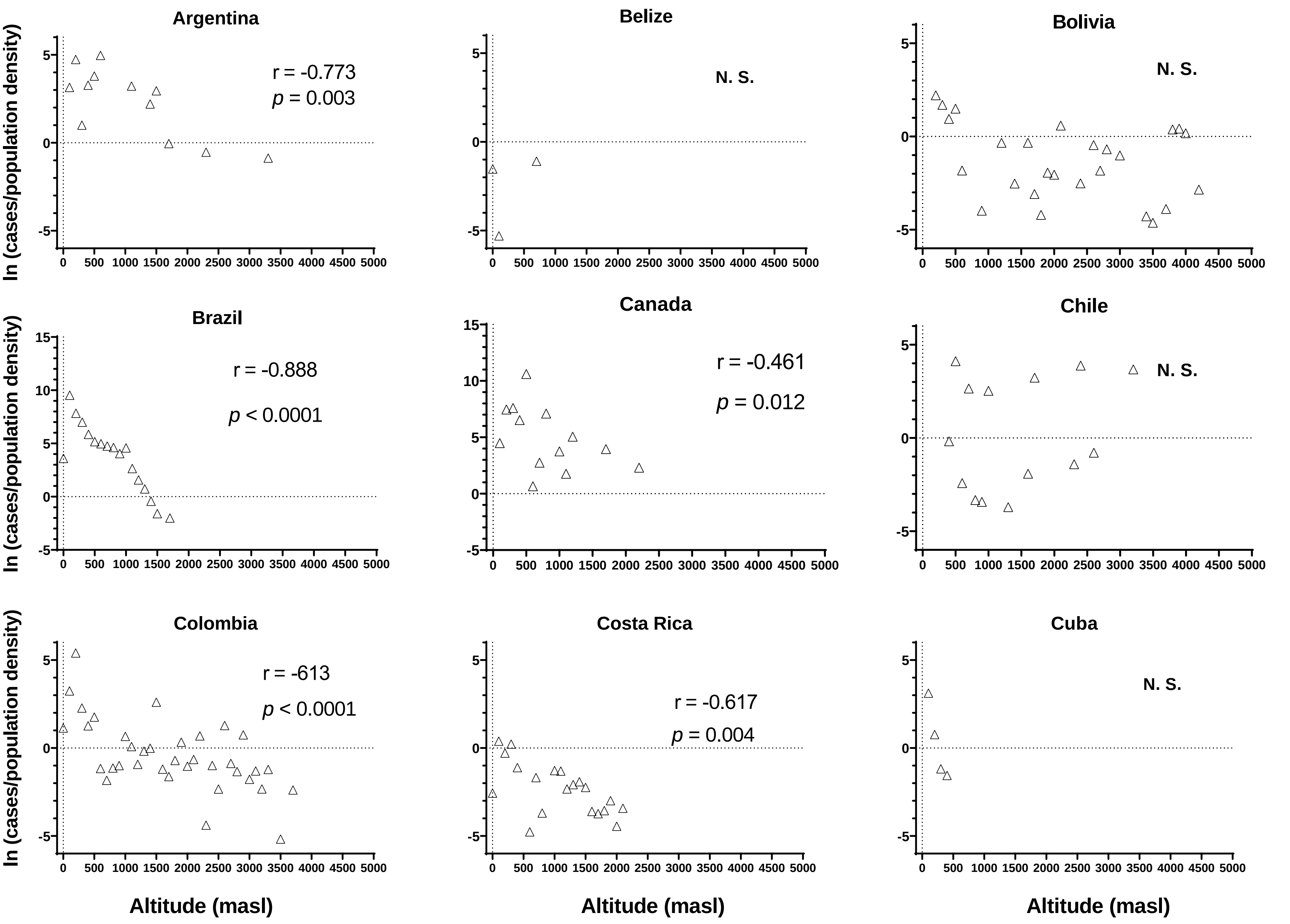

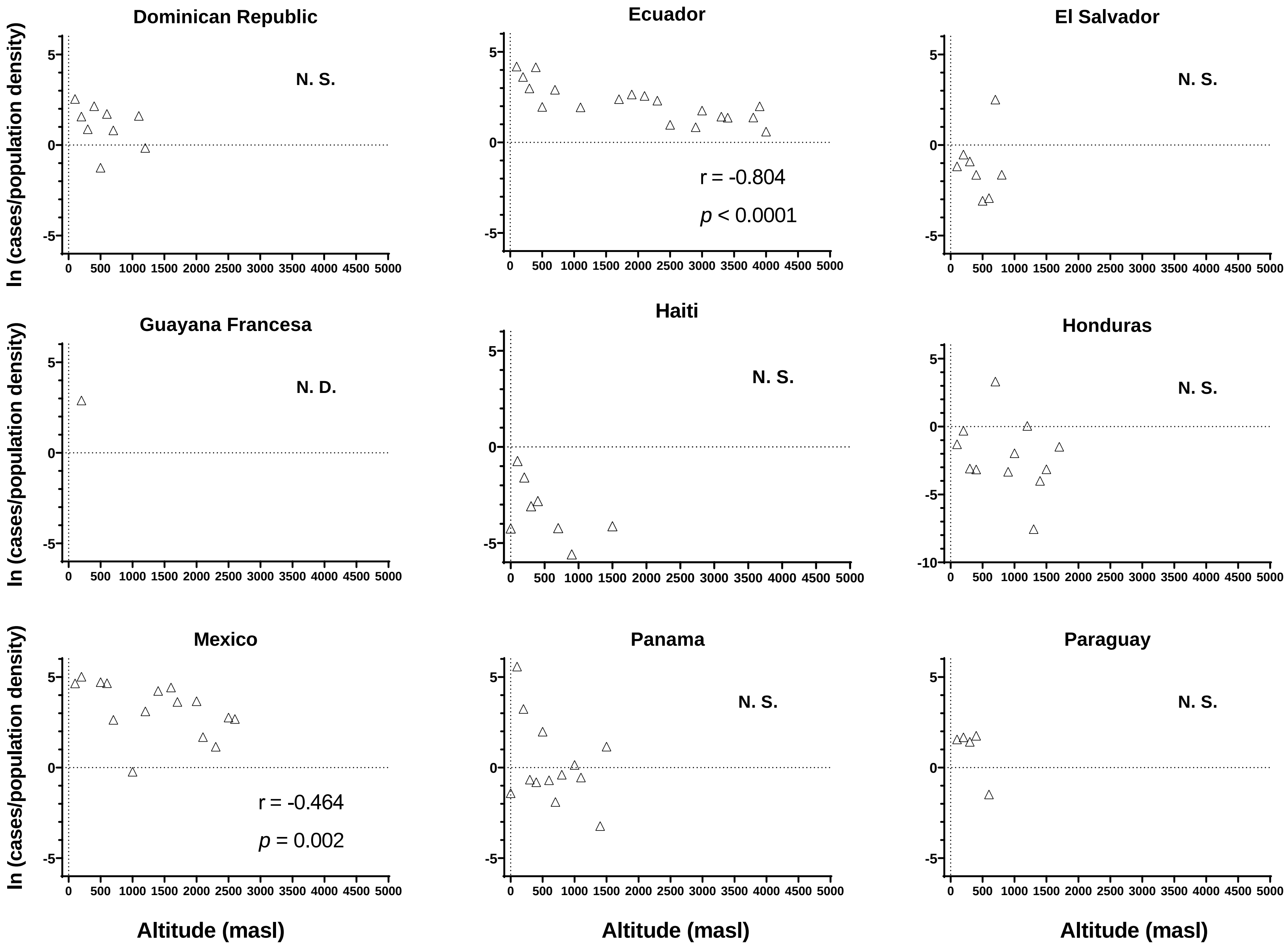

Dominican Republic
N. S.
Ecuador
r = -0.804
p < 0.0001
El Salvador
N. S.
Guayana Francesa
N. D.
Haiti
N. S.
Honduras
N. S.
Mexico
r = -0.464
p = 0.002
Panama
N. S.
Paraguay
N. S.
ln (cases/population density)
Altitude (masl)

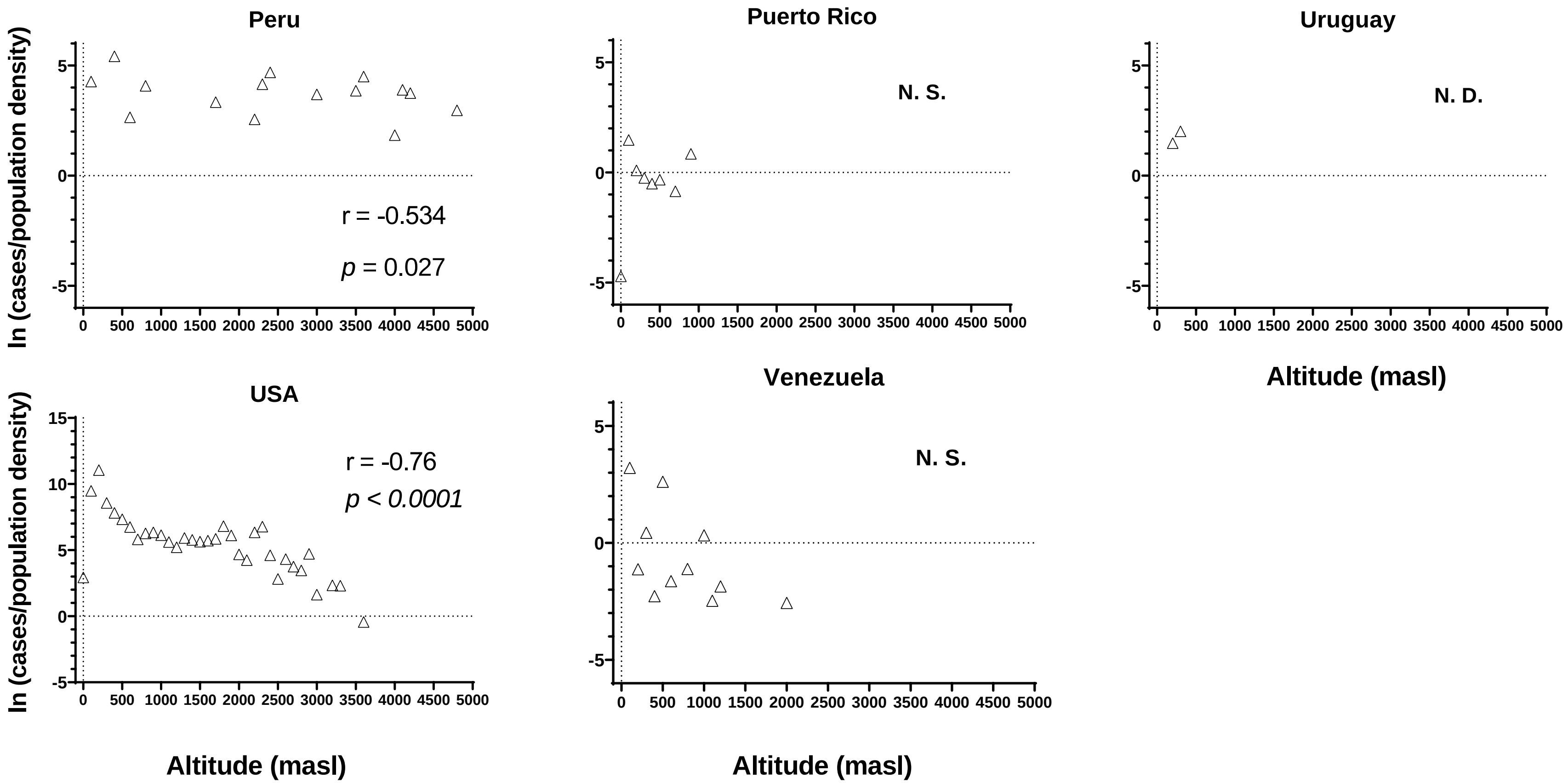

Peru
r = -0.534
p = 0.027
Puerto Rico
N. S.
Uruguay
N. D.
USA
r = -0.76
p < 0.0001
Venezuela
N. S.
ln (cases/population density)
Altitude (masl)
