# Supplementary material for: Decreased incidence, virus transmission capacity, and severity of COVID-19 at altitude on the American continent
Source: PLoS One. 2021 Mar 29;16(3):e0237294. doi: 10.1371/journal.pone.0237294 (PMC8006995; doi:10.1371/journal.pone.0237294)
Supplement: Attachment — Submitted filename: Response to Reviewers.pdf [file pone.0237294.s005.pdf]

UNIVERSITÉ
LAVAL

*Département de pédiatrie*

Ms. No. PONE-D-20-23585

# Decreased incidence, virus transmission capacity, and severity of COVID-19 at altitude on the American continent

## By Arias-Reyes *et al*.

We thank the Editor and the Referees for their important remarks that helped to upgrade the quality of our manuscript. We were pleased to see that the referees found this manuscript technically sound, statistically rigorous, and well written and presented. We wish to respond to your comments as follows:

## JOURNAL REQUIREMENTS:

**1. Please ensure that your manuscript meets PLOS ONE's style requirements, including those for file naming.**

The style of the manuscript meets the style requirements of PLOS ONE.

**2. In ethics statement in the manuscript and in the online submission form, please provide additional information about the database used in your retrospective study. Specifically, please ensure that you have discussed whether all data were fully anonymized before you accessed them and/or whether the IRB or ethics committee waived the requirement for informed consent. If patients provided informed written consent to have their data used in research, please include this information.**

The information from the databases has been included in the manuscript and in the submission form. We confirmed that the data was completely anonymized before accessing it.

**3. Please ensure that you refer to Figure 3 in your text as, if accepted, production will need this reference to link the reader to the figure.**

Figures 2 and 3 were reorganized. Former Figure 3: "Less COVID-19 cases occur above 1,000 masl in the American continent" is Figure 2 in the revised version of our manuscript. The current Figure 2 was properly introduced in the text.

The reference to former Figure 2, Figure 3 in the revised version of our manuscript: "The infection rate of SARS-CoV-2 is decreased above 1,000m of altitude" has been changed.

**4. We note that [Figure(s) 3] in your submission contain [map/satellite] images which may be copyrighted. All PLOS content is published under the Creative Commons Attribution License (CC BY 4.0), which means that the manuscript, images, and Supporting Information files will be freely available online, and any third party is permitted to access, download, copy, distribute, and use these materials in any way, even commercially, with proper attribution. For these reasons, we**

Centre de recherche de l'Institut Universitaire
de Cardiologie et de Pneumologie de Québec
2725 Chemin Ste-Foy,
Québec, QC
G1V 4G5

(418) 656-8711 Poste 8034
Télécopieur: (418) 656-4602
jorge.soliz@crchuq.ulaval.ca

**cannot publish previously copyrighted maps or satellite images created using proprietary data, such as Google software (Google Maps, Street View, and Earth). For more information, see our copyright guidelines: http://journals.plos.org/plosone/s/licenses-and-copyright.**

- Figure 2 (former Figure 3) was created using QGIS 3.14 without using any copyrighted maps or satellite images.
- Geographic data was obtained using the OpenCage Geocoding API which uses open data sources. A full list of data sources used by this API are listed here: https://opencagedata.com/credits
- Altitude data was retrieved from Worldclim 2.0 data base, which explicitly authorizes its open use for research and related activities (https://worldclim.org/data/index.html).
- Population density data was extracted from the dataset created by the Center for International Earth Science Information Network - CIESIN - Columbia University, and it is completely open for any use as stated in: 2018http://www.ciesin.org/documents/CIESINDataPolicy.pdf
- Data of COVID-19 cases were obtained from the official public government sources of each country. All of them are open.
- All the figures and datasets linked to the manuscript are hosted in the repository "figshare" under Creative Commons Attribution License (CC BY 4.0).

**5. We note you have included a table to which you do not refer in the text of your manuscript. Please ensure that you refer to Table 1 in your text; if accepted, production will need this reference to link the reader to the Table.**

Table 1 is now correctly referenced in the introduction section (pg. 4; Ln.68) of the revised version of our manuscript.

## REVIEWER #1 COMMENTS

We thank this referee for his important observation and remarks that helped us improve our manuscript. We wish to respond to his comments as follows:

**Comment 1.** *"One would expect a estimation of location-specific reproduction numbers or infection/disease incidence and infection or case fatality risks when comparing infection rates and disease severity. Instead, a number of rather complex parameters are calculated, without explaining why they are calculated that way."*

**Answer.** All statistical analyses in this work were performed based on classical epidemiological statistics. To do this, we were advised by the epidemiological research center of Laval University.

In brief, in this manuscript we made two types of analyses:

Centre de recherche de l'Institut Universitaire
de Cardiologie et de Pneumologie de Québec
2725 Chemin Ste-Foy,
Québec, QC
G1V 4G5

(418) 656-8711 Poste 8034

Télécopieur: (418) 656-4602
jorge.soliz@crchuq.ulaval.ca

1) Statistical, at population level: To test whether there is an effect of altitude over the incidence of COVID-19.

2) Epidemiological: To evaluate whether the transmissibility and severity of SARS-CoV-2 were affected in highlands.

Our results showing positive correlations between altitude and COVID-19, supported by the significant difference in COVID-19 incidence between locations above and below 1,000 masl (ANOVA), show a clear effect of altitude on COVID-19.

Next, at epidemiological level we calculated the "death-to-case ratio" and the "% of recovered patients". These are the recommended statistical analyzes when the information treated (as the one in the current pandemic) is limited by the quality of the available data. Indeed, most epidemiological parameters calculated come from data series with timely registers, which, even to date, are not fully available for most American countries (usually, only total numbers were reported). Furthermore, the calculation of additional classical parameters, such as the "Infection fatality risk" (also known as Infection fatality rate - IFR) is not possible since IFR is defined as *the risk of death among all infected individuals including those with asymptomatic and mild infections (Yang et al., 2020),* and this information will be complete only when the pandemic ends. In consequence, in the section "3.4 The severity of COVID-19 is reduced in highlands compared to lowlands" we declare that due to this unfeasibility "*we evaluated the differences in the percentage of recovered patients (from the total reported cases) and in the death-to-case ratio (deaths/total reported cases) as indicators of the recovery rate and the IFR*"

Finally, in the revised version of our manuscript, we have included the calculated values of the basic reproduction number ($R_0$) for the lowlands and highlands of the five countries we analyzed in this section in Table 3. As can be seen, consistently $R_0$ values in highlands are lower than in lowlands.

**Comment 2.** *"For instance, incidence is expressed as the natural logarithm of the number of reported cases divided by population density."*

Based on epidemiological statistics, the explanation for this is that:

1. Being SARS-CoV-2 a respiratory virus, it has been suggested that it is more easily transmitted between people in more densely populated places. In this work, we clearly show that there is a significant correlation between the population density and the incidence of COVID-19 (S2). Furthermore, assuming that the high-altitude settlements are less densely populated than the lowlands, it is necessary to normalize the number of cases at each location by the corresponding population density. Classically, the incidence of pathology is expressed as "number of cases per 100,000 inhabitants", however, this parameter does not reflect the effect of population density. For further explanation, please read the response to comment 9.

2. The normalization of the incidence of COVID-19 (# of cases/population density), results in very dispersed values. That is, overpopulated cities with few cases will have very small normalized values (white cells), while less dense cities with many cases will result in very high normalized values (grey cells). See the following example:

Centre de recherche de l'Institut Universitaire
de Cardiologie et de Pneumologie de Québec
2725 Chemin Ste-Foy,
Québec, QC
G1V 4G5
(418) 656-8711 Poste 8034
Télécopieur: (418) 656-4602
jorge.soliz@crchuq.ulaval.ca

UNIVERSITÉ LAVAL

Département de pédiatrie

| City | Province/State | Country/Region | Cases | Pop_den | #Cases/Pop_den |
|---|---|---|---|---|---|
| Tibas | San Jose | Costa Rica | 25 | 13463.556 | 0.001856864 |
| Barra do Turvo | São Paulo | Brasil | 5 | 3412.5 | 0.001465201 |
| Montgomery | Arkansas | USA | 1 | 97.49 | 0.010257105 |
| Durham | Ontario | Canada | 1358 | 1.58140 | 858.7327684 |
| Lima | Lima | Peru | 74037 | 272.4 | 271.7951542 |
| McKinley | New Mexico | USA | 2192 | 5.19 | 422.3506744 |

In this type of dispersion, a logarithmic fit of the data is recommended to facilitate its analysis.

**Comment 3.** *"Separately, a SEIR model is built, but it is unclear why, and what outcome measure this had to provide. Moreover, it is unclear how the number of infections has been estimated as part of the SEIR model, or if that was deducted from it."*

**Answer.** A better explanation of this analysis has been included in the "Results" section of the revised version of our manuscript (pg. 13-14; Ln. 271-276).

First, we used SEIR models to replicate (mathematically) the real data reported for the lowlands (<1,000 masl) and highlands (>1,000 masl) of Argentina, Bolivia, Colombia, Ecuador, and Colombia. To do so, we calculated the number of "Susceptible", "Exposed", "Infected", and "Removed" individuals (from the date the first case was reported in the corresponding country until May 23) using the theoretical parameters (initial number of infected, number of exposed subjects, contact rate, recovery rate, and the rate at which exposed individuals become infected) as described in the methods section. Next, we adjusted such parameters of the model to match the real reported numbers of "Infected" people for the highland and lowland populations separately. In doing so, in the mathematical model, we "played" with the "transmission rate" in such a way that they allow the most faithful reproduction of the epidemiological curves observed in the highlands and lowlands. For the five above mentioned countries, we found that using lower values of transmission rates reproduce better the real data for highland populations. On the contrary, higher values of transmission rates reproduced better the real data for lowland populations.

**Comment 4.** *"For severity, a 'death-to-case' ratio and pct recovered patients were calculated, rather than an infection fatality risk, which would have been more appropriate."*

**Answer.** Please see the answer provided for Observation 1.

**Comment 5.** *"Moreover, it is unclear at what stage during the outbreak these were estimated (during the exponential increase? which would overestimate the number of cases as compared to deaths), and it seems like no reporting+symptom to death time lag (delay between symptoms and death, and a delay in reporting deaths) were considered."*

Centre de recherche de l'Institut Universitaire
de Cardiologie et de Pneumologie de Québec
2725 Chemin Ste-Foy,
Québec, QC
G1V 4G5

(418) 656-8711 Poste 8034

Télécopieur: (418) 656-4602
jorge.soliz@crchuq.ulaval.ca

**Answer.** As previously mentioned, the data analyzed correspond to those collected from the date of notification of the first case (for each country) until May 23. Regarding the epidemiological analyses to which the reviewer refers, in Argentina, Bolivia, Colombia, Ecuador, and Peru this period includes early stages of the exponential increase phase in the lowlands, but not in the highlands (with exception of Colombia), where cases remained low passed May 23 (Fig 3).

The delays between the notification of symptoms and deaths and the delay in the notification of deaths are not available, much less at the required geographical level (state/province/departamento). Therefore, it is not possible to relate these data to altitude. As far as we know, even today, these data, with such geographic resolution, are not available. Furthermore, the Pan American Health Organization (PAHO) informed us that they do not have this information in their databases either. Although the referee's observation is pertinent, it will not be possible to carry out this type of analysis until the pandemic is over and all the data is completed in detail and made available.

**Comment 6.** *"Most importantly, potential third factors, which could importantly confound the association between altitude and incidence or severity, such are differences in population age structure between populations living in places with higher or lower altitude, are not taken into account."*

**Answer.**

We thank this referee for this important observation. Indeed, we mentioned this limitation in our work in the discussion section of our manuscript (pg. 20; Ln. 419-423). It is important to note, however, that to date, with few exceptions, official COVID-19 data sources (i.e. governments, health agencies, and research institutes), only provided information on the total number of cases (cumulative), total deaths, and in some cases the total number of recoveries. So far, no institution, in any country, has provided information on more detailed epidemiological factors, such as age structure, comorbidities, or sex of those infected.

Please note that with all these limitations, we were able to evaluate and analyze the COVID-19 incidence data from more than 8,000 locations corresponding to 23 countries within the American continent. In this sense, we believe that our reports have great epidemiological value and will serve as a basis for the development of new studies, we hope that they will be more complete and detailed.

**Comment 7.** *"For Figure 1, it is unclear why 4 figures are provided, and to what extent they differ. Are b, c and d just zooms of the first figure? why are some points which were shown in fig 1a missing in 1c?"*

**Answer.** We thank the reviewer for this important observation. In effect, we put an incorrect graph on panel c in Figure 1. The correct graphic is now in place.

Panel **a** shows the correlation between altitude and the incidence of COVID-19 considering the entire altitudinal range (0 - 4,800 masl) of locations with COVID-19 cases in the American continent. In this figure, the points (open triangles) represent the summatory of the incidence every 100 meters of altitude. This graph is important because it shows an effect of altitude on the incidence of COVID-19.

Panel **b** shows the same data as panel **a** but broken down for each altitude (the data without grouping every 100 meters of altitude). This graph is important because it shows that there is a significant cut in the incidence of COVID-19 at 1,000 meters above sea level (shown by the red dotted line).

Centre de recherche de l'Institut Universitaire
de Cardiologie et de Pneumologie de Québec
2725 Chemin Ste-Foy,
Québec, QC
G1V 4G5

(418) 656-8711 Poste 8034
Télécopieur: (418) 656-4602
jorge.soliz@crchuq.ulaval.ca

To make this cut of COVID-19 incidence at 1,000 meters more evident, we carried out the analyzes showed in panels **c** and **d**, which evidence that there is indeed no incidence of altitude up to 1,000 meters (Panel **c**) and that the effect of altitude begins from 1,000 meters up (Panel **d**). These last two graphs are important because they statistically show that the effect of altitude begins at 1,000 meters.

A clearer description of this figure was included in the corresponding legend of the corrected version of our manuscript.

**Comment 8.** *"For Figure 2, it is unclear what the percentages stand for, and what the different dashed lines stand for. The figure is described as 'effect', but it is a mere comparison of two observations."*

**Answer.**

Note that figure 2 above is the current figure 3.

The percentage values are the "transmission rate" values that are used to theoretically calculate the numbers of susceptible, exposed, infected, and removed people over time with the SEIR model for COVID-19. As can be seen in this figure, to make a representative theoretical calculation of these curves, different "transmission rate" values are necessary for the lowlands and the highlands. As such, if we use the same "transmission rate" for lowlands and highlands, the theoretically calculated graphs would not reflect the reality (solid black lines). Thus, the percentage values in blue are the "transmission rate" that is suitable for modelling the lowland data (dotted lines in blue). Instead, these values in the highland figures show how the same "transmission rate" does not model the real data from highlands. On the other hand, the percentage values in red are the "transmission rate" that is suitable for modelling the data in highlands (lines dotted in red). These graphs are important because they show that to model the highland data of COVID-19 infection, lower "transmission rates" of the virus are required than those required for modelling the data of lowlands. Biologically, this implies that the probability of transmission of the SARS-CoV-2 virus is reduced in the highlands compared to lowlands.

In the revised version of our manuscript, we include a more detailed explanation of this figure in the corresponding legend.

**Comment 9.** *"For Figure 3, it is unclear to me why population density would not already be taken into account when calculating incidence in a conventional way. I would be very interested to know why you use a natural logarithm and divide by km2."*

**Answer.** Incidence, traditionally reported as number of cases/100,000, inhabitants is mathematically limited by the dividend to the number of total population in a zone, without considering the total area ($km^2$) of such zone. For a better clarification see the following example comparing two fictitious cities:

Scenario 1: Only the number cases is different between the two cities.

Scenario 2: Only the population is different between the two cities (and this changes the population density).

Scenario 3. Only the area is different between the two cities (and this changes the population density).

Centre de recherche de l'Institut Universitaire
de Cardiologie et de Pneumologie de Québec
2725 Chemin Ste-Foy,
Québec, QC
G1V 4G5

(418) 656-8711 Poste 8034

Télécopieur: (418) 656-4602
jorge.soliz@crchuq.ulaval.ca

UNIVERSITÉ LAVAL

Département de pédiatrie

Scenario 4. The area and the number of cases are different between the two cities.

| | | Population | Area (km$^2$) | Pop. Density (people/km$^2$) | COVID-19 cases | Cases/100,000 people | Cases/pop. Dens. |
|---|---|---|---|---|---|---|---|
| Scenario 1 | City A | 500,000 | 100 | 5000 | 50 | 10 | 0.01 |
| | City B | 500,000 | 100 | 5000 | 10 | 2 | 0.002 |
| Scenario 2 | City A | 500,000 | 100 | 5000 | 50 | 10 | 0.01 |
| | City B | 250,000 | 100 | 2500 | 50 | 20 | 0.02 |
| Scenario 3 | City A | 500,000 | 100 | 5000 | 50 | 10 | 0.01 |
| | City B | 500,000 | 50 | 10000 | 50 | 10 | 0.005 |
| Scenario 4 | City A | 500,000 | 100 | 5000 | 10 | 2 | 0.002 |
| | City B | 500,000 | 50 | 10000 | 50 | 10 | 0.005 |

In scenarios 1 and 2, both ways to calculate incidence (Cases/100,000 people and Cases/population density) are equivalent. However, in scenarios 3 and 4, when the population density is different between the two cities due to changes in the area, normalizing the number of cases by population density results in a higher value of incidence (in comparison with the other method), thus, revealing locations where the small number of COVID-19 cases is related with low population densities. Such situation has been suggested to happen in rural settlements (particularly in high altitudes), where people live far away from each other.

Regarding the logarithmization, please see the answer 2.

**Comment 10.** *"For Figure 4, comparing countries, stating quarantine measures were comparable, does not seem an appropriate way to answer your research question, for many reasons including some stated above (pop age structure, reporting differences, etc.)"*

**Answer.** Figure 4 shows the number of infected people estimated for highland populations of Argentina, Bolivia, Colombia, Ecuador, and Peru, in a scenario in which quarantines would not be applied in these countries. Figure 4 presents the real (reported) data in the black-dotted line, the blue line represents the data modelled (SEIR models) to emulate the real data and the red line represents the modelled data using a higher value of "frequency of interaction" (a parameter of SEIR models) to simulate the absence of a quarantine. The values of frequency of interaction used to calculate the blue and red lines are detailed in the methods section.

As stated in the main text (pg. 20; Ln. 408-414), the intention of this analysis is to show that social isolation measures are crucial to reduce the number of infected people regardless of altitude. This is important because the readers of this report could interpret our results as that quarantine and social

Centre de recherche de l'Institut Universitaire
de Cardiologie et de Pneumologie de Québec
2725 Chemin Ste-Foy,
Québec, QC
G1V 4G5

(418) 656-8711 Poste 8034

Télécopieur: (418) 656-4602
jorge.soliz@crchuq.ulaval.ca

isolation measures, especially in highlands, are not necessary to decrease the transmission of the virus.

Moreover, our modelled data (blue lines) emulates well the numbers of infected people for each of the five countries analyzed regardless of the omission of more detailed epidemiological parameters as those mentioned by the reviewer. This shows that all those parameters, although remarkable, are not determinant to reach the conclusions obtained in this work. In any case, as mentioned above, such additional parameters are not available, especially at the level of geographic resolution required for this work, and it is possible that these data will be available for analysis one or two years after the end of the pandemic.

## REVIEWER #2 COMMENTS

We were pleased to see that this referee stated that our manuscript is informative, interesting, and well written and presented.

**Comment 1.** *The article presents epidemiological data as of 23rd May. Authors may add some more recent literature supporting their finding (if any!) and any other contrasting report (if any!)*

**Answer.** The revised version of our manuscript includes, in the discussion section, the references of recently published works (after the initial presentation of this manuscript).

## BIBLIOGRAPHY CITED IN THIS LETTER

Yang, W., Kandula, S., Huynh, M., Greene, S. K., Van Wye, G., Li, W., . . . Olson, D. (2020). Estimating the infection-fatality risk of SARS-CoV-2 in New York City during the spring 2020 pandemic wave: a model-based analysis. *The Lancet Infectious Diseases*.

On behalf all authors

Dr. Jorge Soliz

Centre de recherche de l'Institut Universitaire
de Cardiologie et de Pneumologie de Québec
2725 Chemin Ste-Foy,
Québec, QC
G1V 4G5

(418) 656-8711 Poste 8034

Télécopieur: (418) 656-4602
jorge.soliz@crchuq.ulaval.ca
