# Supplementary material for: Decreased incidence, virus transmission capacity, and severity of COVID-19 at altitude on the American continent
Source: PLoS One. 2021 Mar 29;16(3):e0237294. doi: 10.1371/journal.pone.0237294 (PMC8006995; doi:10.1371/journal.pone.0237294)
Supplement: Attachment — Submitted filename: Response to Reviewers.pdf [file pone.0237294.s006.pdf]

UNIVERSITÉ LAVAL
Département de pédiatrie

Ms. No. PONE-D-20-23585R1

Decreased incidence, virus transmission capacity, and severity of COVID-19 at altitude on the American continent

By Arias-Reyes *et al*.

We thank the Referee for her important remarks that helped to upgrade the quality of our manuscript. We were pleased to see that the referee found our manuscript to be transparent about our methods and process of thought. We wish to respond to your comments as follows:

REVIEWER #3 COMMENTS

1. SUMMARY

This study aims to evaluate the impact of altitude on the manifestation of SARS-CoV-2. In particular, the correlation between altitude and incidence of COVID-19, it's severity, and the transmissibility of SARS-CoV-2. The authors focus on the countries of the American continent. Overall, the authors are transparent about the methods used and their thought process. However, the manuscript can do with some good editing. More specifically, without wanting to come across mean or rude, it appears the manuscript is not written by someone with a statistical background and can do with more clarification (see below for more detail) as well as a rewrite, e.g. pp 6 line 107-115, terms like "the analysed variable" are not conventional terms to use, rather something along the lines of "dependent variable". Also, sections in the results are better suited in the discussion and/or methods. Moreover, the manuscript comprises quite some repetition in methods used (e.g. normalised and logarithmatised is repeated many times unnecessarily). Finally, and perhaps more importantly, I have some doubts about the methods employed and interpretation of the results, among which the methods used to assess the respective transmissibility in the 'highlands' vs 'lowlands'.

– Pp6 lines 114-115: The text has been changed, now it reads: "The dependent variable was…"

– The text "normalised and logarithmized" was removed from the manuscript in several parts to avoid repetition. Also, the following text has been added in the Methods section:
Pp 5 lines 104-105: "These data (referred as the number COVID-19 cases) were used for all the analyses unless stated otherwise."

– Some sentences in the results sections have been moved to the methods section according to the suggestions of the reviewer (see minor comments).

– Regarding the methods employed to assess the transmissibility, please see the answer to the comment 2.5.

2. MAJOR COMMENTS

2.1. The authors use the Pearson correlation coefficient to assess among others the linear relationship between altitude and incidence rates and incidence and population density. First of all, this test is valid when both variables of concern are normally distributed. Could the authors please

confirm whether they assessed normality in their variable distributions? Otherwise a non-parametric test might be more suited.
Secondly, R2 is listed along side the estimated pearson's correlation. This could be me, but I would say reporting pearson's r is more common. Can the authors confirm what is reported is the R2 and why? Now more importantly, the authors report on the significance of their correlation between COVID-19 incidence and population density.

Yes, both variables, altitude, and COVID-19 cases, had normal distributions (Anderson-Darling test $A2_{Altitude}$=0.458; $p_{Altitude}$=0.251; $A2_{COVID\text{-}19\_cases}$= 0,633; $p_{COVID\text{-}19\_cases}$ =0.092). The values of Pearson's "r" are now reported instead of R^2 in both, the main text, and the figures.

2.2. Although I have nothing against normalising the result by population density, I doubt relying on merely a significant p-value with such a low R2 provides the right 'prove' to do so (this might relate to a high sample size, but as listed in the minor comments, it is a bit unclear to me which test is fitted to which data). I think this is also confirmed by the high variance observed in the correlation between these two variables in Figure S2. Perhaps better to explain rational for normalising incidence by population density in the methods and leave out 3.1.

We appreciate the suggestion of the reviewer. We have included the rationale for normalizing the data of COVID-19 cases by population density in the methods section:

"The number of COVID-19 cases by location was normalized by population density (inhabitants per square kilometer), in accordance with previous studies that demonstrated a positive correlation between the population density and the number of COVID-19 cases [23-26]."

Also, the section 3.1 of the results was removed as well as Figure S2.

2.3. I find the authors conclusions more concerning for table 2, where significant p values go alongside with a wide range of R2 values. Explanation is in part covered in the discussion section pp 18, but this is for the countries where no correlation is found. I think this could be done more elaborate, among which how quality of passive surveillance could affect the findings in terms of strength of the correlation.

We thank the reviewer for this comment and suggestion. Firstly, table 2 has been modified to include "Pearson's r" instead of R squared following a previous suggestion from the reviewer. Secondly, in Table 2 we show how the incidence of COVID-19 is negatively correlated with altitude even when analyzing American countries individually. These findings correspond well with our results at whole-continent level (correlation and Random block design ANOVA). Since the results are consistent, we believe that the most important matter to discuss, is why some countries with large populations living above 1,000 masl do not show significant correlations. We agree with the observation of the reviewer saying that the strength of correlation may be affected by the quality of passive surveillance, however, this is a topic challenging to study in the Americas since health policies are very heterogeneous among countries. Also, the information regarding surveillance policies during the COVID-19 pandemic is not

always accessible or trustable in these countries, thus any discussion in this regard may result speculative.

2.4. The authors are speaking of "the SEIR" model, but in fact, SEIR model structures can involve a multitude of assumptions and parameters encompassing these assumptions. As a result, I have some difficulty assessing the validity of the findings regarding the evaluation of the virus transmission rates.

2.4.1. Therefore, first of all "an SEIR model structure" would be more appropriate on pp6 line 122.

The correction has been made, now the text reads: "A SEIR model (Susceptible - Exposed - Infectious - Removed) was used to…"

2.4.2. Also, a listing of the differential equations either in the methods section or the supplementary material would be useful to provide the reader transparency in what model parameters and assumptions were incorporated. E.g. what is assumed regarding the transmission rate of asymptomatic individuals? Non infectious at all? What fraction of the population is assumed to remain asymptomatic?

The equations, parameters, values, and the sources of the values used in our model are now described in the supplementary material S3. Also, the following text has been added in the methods section:

"We set the values of interaction frequency = 8.1 [28], infectious period = 7.5 days, and incubation period = 6 days [29]. Asymptomatic individuals were considered as non-infectious. Recovered individuals were considered to be immune to reinfection. The size of the population was considered unchanged during the modelled time lapse."

2.4.3. Also, it is good practice to list the parameters values used as well as their source. Therefore, I would argue for a table in the supplementary material.

Please see the response to the comment 2.4.2.

2.4.4. Moreover, the interaction frequency needs explaining. A more commonly used term I think is "contact rate". The authors refer to beta as their contact rate, but this would more commonly be coined "the effective contact rate" (which can be denoted as a product of contact rate and probability of infection upon contact).

We named the terms as described in (Abadie, Bertolotti, & Arnab, 2020). Indeed, in our manuscript beta is referred as the "contact rate" according to the following equation:

$$\beta = interaction\ frequency\ among\ individuals * probability\ of\ transmission\ of\ the\ disease$$

Where:

β: Contact rate

The description of the parameters is now included in S3.

Centre de recherche de l'Institut Universitaire
de Cardiologie et de Pneumologie de Québec
2725 Chemin Ste-Foy,
Québec, QC
G1V 4G5

(418) 656-8711 Poste 8034

Télécopieur: (418) 656-4602
jorge.soliz@crchuq.ulaval.ca

2.5. More importantly, heterogeneity in transmission of SARS-CoV-2 has been outspoken (e.g. https://wellcomeopenresearch.org/articles/5-67/v3, https://doi.org/10.1101/2020.08.09.20171132), which has among others been associated to heterogeneities in age-specific contact patterns. Therefore, estimating an R0 based on an average contact rate, probability of transmission as well as infection period is not a very accurate representation of SARS-CoV-2 dynamics. Importantly, quantifying differences between country-level altitude, by "playing around" with the transmission rate does not come across methodologically sound and could, at minimum, do with a formal fitting procedure.

We calculated R_0 values as they were requested by another reviewer. Moreover, as described in the text (pp 6 line 121), the data we analyzed was taken during a period of strict quarantines in Argentina, Bolivia, Colombia, Ecuador, and Peru, when the mobility of people was drastically reduced. So, it is fair to assume that the heterogeneity in contact rate among age groups also decreased.

Our models were originally adjusted using the least squares method, however, attending the accurate observations of the reviewer, we have adjusted our models again by using the maximum likelihood method. Also, we have calculated the confidence intervals of the estimated transmission rates. Although the estimated values of probability of transmission differ slightly from those we reported in the original version of our manuscript, the trends and conclusions remain unaltered (except for Colombia).

These procedures together with the new values were added/replaced in the corresponding sections of the manuscript, including Fig 3 and Table 3.

2.6. Moreover, in my opinion the differences between transmission rates in the high and lowlands are hardly convincing. A deterministic model is used, and confidence intervals are missing, but my suspicion is that these would highly overlap.

We appreciate the observation of the reviewer; however, we want to emphasize that we set our SEIR models to match the data of cases officially reported by May 23rd for the highland and lowland regions of each country (dotted lines in Figure 4). In our exercise we aimed to replicate the reported data, not to predict future trends. In this context, a deterministic model should work well. Moreover, as the reviewer can see in Table 3, the confidence intervals calculated for the estimated transmission rates do not overlap.

2.7. What I am also not certain about (but I might have missed it), do the authors vary the "frequency of interaction rate" between high and lowlands? As the SEIR model is 'fitted' to raw incidence, one would expect different frequency of interaction rates in higher than in lower densely populated areas. In a dynamic transmission model where beta, the effective contact rate, is a product of the frequency of interaction and the probability of infection upon contact, the 'fitted' value for the latter will be correlated to the value used for the former. This needs clarification.

As mentioned above, the data we analyzed corresponded to the period between the first reported case for each country (all of them around March 10th) and May 23rd, a period during which strict

UNIVERSITÉ LAVAL
Département de pédiatrie

quarantines were applied in theses countries, therefore, the difference in contact rates between populations with high and low population densities that would occur in regular conditions was drastically reduced.

2.8. In conclusion, I feel rather uncomfortable by the SEIR model used and validity of the findings reported on the transmission rates and R0 estimates between highlands vs lowlands. I doubt these findings should be part of the manuscript.

We are aware that using a deterministic model implies limitations, however, we believe that the differences we found in transmission rates between highlands and lowlands are very clear. This is supported by the small confidence intervals we found for the estimated parameter. So, even if the values we estimated for the transmission rates and R_0 are not precise, the epidemiological trends should not be considerably different. We are convinced that this part of our work offers important support to the hypothesis of an attenuated effect of COVID-19 in high regions.

2.9. Estimate for severity is based on the recovered to case ratio and recovery rates. For clarification, what do the deaths represent in the death to case ratio? The national reported covid deaths? Excess mortality? As I am not entirely sure why there should be such a gap between 1-fraction of of recovered patients (recovered/total cases) and death to case ratio (deaths/total cases).

Deaths represent the total deaths reported for the corresponding altitude group: i.e., above 1,000 and below 1,000 masl. Accordingly, the recovered patients represent the total recoveries reported for the corresponding altitude group. Thus, the gap the reviewer mentions, represents the fraction of active cases, those which are not dead nor recovered either.

The text in section 2.4 of the manuscript has been modified accordingly:

"The death-to-case ratio and the percentage of recovered patients ([recovered patients/reported cases] * 100) for each country (except Ecuador) were calculated using the data from the last 10 days evaluated (from May 13th to 23rd) for the populations above and below 1,000 masl in two separate pools. The number of deaths and recoveries used to calculate these parameters are the summatory of the values reported for all the populations above and below 1,000 masl."

2.10. Regardless, both are very likely sensitive to underreporting (i.e. the higher underreporting, the higher the death-to-case ratio). This is the reason why the authors evaluate the underreporting of cases in high and lowlands. The authors conclude that the non-significant differences observed in death-to-case ratio between high and lowlands could be explained by differences in in undiagnosed cases (76% for highlands vs 73% for lowlands). Estimating confidence intervals based on mean and SD will, I think, show that these estimates overlap, i.e. revealing that this might not necessarily explain the non-observed difference. Even if there was a true difference in underreporting between high and lowlands, why would one expect a difference in underreporting based on altitude? Please clarify and what could be an alternative explanation. In particular why

Centre de recherche de l'Institut Universitaire
de Cardiologie et de Pneumologie de Québec
2725 Chemin Ste-Foy,
Québec, QC
G1V 4G5

(418) 656-8711 Poste 8034

Télécopieur: (418) 656-4602
jorge.soliz@crchuq.ulaval.ca

UNIVERSITÉ
LAVAL
Département de pédiatrie

there is a difference in %recovered but not in death to case ratio (but as stated, I don't fully understand the difference).

The reviewer is right, both indicators are very sensitive to underreporting, specially considering the limitations that these countries had for testing people during the first months of the pandemic.

The underreporting would be directly associated with a higher proportion of asymptomatic, mild, and moderate cases occurring in the highlands as a result of the lower severity of the disease in these regions. Due to the scarcity of tests, during the first months of the pandemic, diagnosis was favoured to people showing clear symptoms or reporting recent contact with infected subjects. Asymptomatic, mild, and a fraction of moderate cases would not be diagnosed.

Mortality rates may be indicators of the access to ICU facilities, opportunity and quality of clinical treatment, and the severity of the disease in severe and critical patients. While the recovery rate may include the effectiveness of out-of-hospital (pharmacological interventions) treatment (most common strategy to treat COVID-19 in Latin America), as well as the factors related with mortality when concerning severe and critical cases. In this context, no differences in mortality rates but a higher recovery rate in the highlands compared to lowlands, suggest again a lower severity, at least in asymptomatic, mild, and moderate cases, of COVID-19 in the highlands.

2.11. Also methods pp 7 lines 149 – 151: COVID severity: These seem to be based on national estimates (npairs = 5). This while the estimates for the correlation between cases and altitude and case numbers are based on a mixture of national, regional and local level data (see minor comments). Why the difference? To what extend does it make sense to use national level data here, why not, similar to the analyses correlating altitude with incidence, on a more granular level?

We wanted to evaluate this data using a paired design, so we could eliminate inter-country effects. Considering this, it would be very difficult (if not impossible) to pair, objectively, states from the lowlands with states from the highlands. In consequence, we decided to pool the data from the lowland states and the highland states in two separate datasets for each country (5 countries), then we used these pairs to evaluate the differences in recovery and mortality.

As stated in the manuscript, these analyses were made at state level.

2.12. Discussion pp 19: This seems to elaborate quite far on what is covered in the manuscript. Virus transmission capacity under different altitudes I think is what the authors want to elude to, but it now comes across somewhat as a self-citation exercise and covering a topic the authors is probably familiar with. Suggest to cut and/or shorten.

We appreciate the comments of this reviewer. However, we think that, since a limited amount of literature is available on the subject, it is important to report as much as possible our epidemiological findings and make links with likely theoretical and physiological explanations to help in the building of a better understanding of the particular behaviour of COVID-19 in highlands.

Centre de recherche de l'Institut Universitaire
de Cardiologie et de Pneumologie de Québec
2725 Chemin Ste-Foy,
Québec, QC
G1V 4G5

(418) 656-8711 Poste 8034

Télécopieur: (418) 656-4602
jorge.soliz@crchuq.ulaval.ca

3. MINOR COMMENTS

3.1. ***Methods pp 5 line 105: what do the n=51 represent? Is this a mixture of observations on city or province or state or country or departmental level?***

Two changes have been inserted in the text to clarify this point:

– "The number of COVID-19 cases by location (per city/county or per state/province/departamento) was normalized by population density (inhabitants per square kilometer), in accordance with previous studies that showed a positive correlation between the population density and the number of COVID-19 case."

– "The correlation between the number of COVID-19 cases per 100-meters-of-altitude interval and the altitude was analyzed using a Pearson correlation analysis (n= 51)."

3.2. Similar for pp 6 lines 109-110, what do the n's represent here? It seems country-level datapoints, but as the block variable concerns 'country' there must be multiple observations from within the countries included. Moreover, these are estimates all across the whole world, not only the American continent?

This analysis was performed at continental level (23 countries). Each data point represents the number of COVID-19 cases at a certain altitude within one country. These altitudes correspond to each city/county or state/province/departamento (according to the availability of data) where COVID-19 cases were reported. This explains the great number of data points. The text has been modified accordingly:

"The dependent variable was the number of COVID-19 cases (at 2nd or 3rd administrative level and not grouped by altitude intervals); the grouping variable was the altitude (> 1,000 masl or < 1,000 masl)"

3.3. And what about the n's listed in line 111-115 for those countries in the American continent? Please clarify.

These "n" values represent the number of data pairs analyzed in the correlation for each country. The text describing these tests has been moved next to the description of the initial Pearson correlation for clarity.

3.4. For the non-statistical reader, it would be good to refer to "the block variable" in a more intuitive way.

We thank the reviewer for this suggestion. We have changed the sentence for: "…and the blocks were the countries." As we do not see a better way to explain the design. If the reviewer has a better suggestion, we will gladly accept it.

3.5. pp 6 line 107-115, terms like "the analysed variable" are not conventional terms to use, rather something along the lines of "dependent variable".

Centre de recherche de l'Institut Universitaire
de Cardiologie et de Pneumologie de Québec
2725 Chemin Ste-Foy,
Québec, QC
G1V 4G5

(418) 656-8711 Poste 8034

Télécopieur: (418) 656-4602
jorge.soliz@crchuq.ulaval.ca

The text has been changed to: “The dependent variable was the number of COVID-19 cases…”

3.6. “organised by intervals of 100m of altitude”. ‘Categorised’ or ‘grouped by’ might be preferred.

The text has been changed according to the suggestion of the reviewer: “These data were then grouped by intervals of 100 meters of altitude.”

3.7. Pp 6 line 122: The SEIR should be An SEIR. There is not just one SEIR model.

Changes have been made accordingly throughout the text:

Pp 6 line 125 “A deterministic SEIR model (Susceptible - Exposed - Infectious - Removed) was used…”

Pp 13 line 274 “This causes the requirement of a higher infection probability value in our SEIR model to fit…”

3.8. There are certain sections in the result section, that are better suited in the discussion, as these provide interpretation of the results in the context of existing evidence, i.e.:
3.8.1. Results pp 10 line 196 – 197. This should be moved to the discussion.

This section has been removed.

3.8.2. Results pp 12 lines 258-261: This should be moved to discussion.

The text has been modified. Now it reads:

“Taken together, these findings show that a significant decrease in the incidence of COVID-19 starts above 1,000 m of altitude.”

3.9. Also, no need to repeat again that cases were normalised by population density. The same holds for pp 10 lines 203-204. The methods clearly describe what scale was assumed for the correlations, no need to list this again.
This was corrected throughout the text. Also, the following text has been added in the Methods section:
Lines 104-105: “These data (referred as the number COVID-19 cases) were used for all the analyses unless stated otherwise.”

3.10. Methods pp 10 line 207: I think a word is missing in this sentence, but also I don’t fully understand what is meant. I think that beyond 1000 masl, a correlation between altitude and COVID-19 incidence is apparent (and not below) but this is not what it reads.

The reviewer is right, there was a typo in the sentence. The text has been changed accordingly:

Centre de recherche de l’Institut Universitaire
de Cardiologie et de Pneumologie de Québec
2725 Chemin Ste-Foy,
Québec, QC
G1V 4G5

(418) 656-8711 Poste 8034
Télécopieur: (418) 656-4602
jorge.soliz@crchuq.ulaval.ca

"No significant correlation was found for data below 1,000 masl (p=0.568; r= -0.206) (Fig 1c), while a strongly significant correlation between COVID-19 incidence and altitude was obtained for altitudes above 1,000 masl…"

3.11. I also don't understand what the authors have done when they state "repeated correlation analyses performed at altitudes above 800, 1000 etc."

The text has been changed to: "In separate correlation analyses considering data from altitudes above 800, 1,000, 1,500, and 2,500 masl, we confirmed this observation."

3.12. Methods pp 11 lines 219 – 221: Move to methods.

The sentence "The advantage of this type of statistical analysis is that it considers the internal variability of each country in the incidence analysis." Has been moved to methods.

3.13. Figure 1B legend: add "above 1000m"?

The panels in the figure have been reorganized. Now the legends match correctly with the panels.

3.14. Fig 2: It might be the resolution, but I fail to see the blue circles. Should these be 'red circles'?

The reviewer is right. "Blue circles" has been changed for "Red circles" in the legend of Figure 2.

4. TYPOS

4.1. Introduction pp4 line 76: Appears to have referencing non-consistent with the remainder of the article

The reference has been formatted accordingly.

4.2. Introduction: It's not very common to include tables in the introduction. Is this table really needed? Or can it be moved to Supplementary material?

Table 1 has been moved to supplementary material (now it is S1) and the text has been changed accordingly.

4.3. Methods pp 8 lines 160-165: Sentence is not correct.

The sentence has been restructured. Now it reads: "Since health policies in most countries in the American continent restricted the access to COVID-19 tests to people showing clear symptoms of infection or with history of contact with infected people…"

4.4. Methods pp 8 line 167: Ref 28 (worldometer) the correct reference?

Centre de recherche de l'Institut Universitaire
de Cardiologie et de Pneumologie de Québec
2725 Chemin Ste-Foy,
Québec, QC
G1V 4G5

(418) 656-8711 Poste 8034
Télécopieur: (418) 656-4602
jorge.soliz@crchuq.ulaval.ca

UNIVERSITÉ LAVAL
Département de pédiatrie

The reference has been updated using the citation format suggested by the web platform: "Worldometers.info. Coronavirus Death Rate (COVID-19) - Worldometer Dover, Delaware, U.S.A.2020 [19/05/2020]. Available from: https://www.worldometers.info/coronavirus/coronavirus-death-rate/."

5. REFERENCES CITED IN THIS LETTER

Abadie, A., Bertolotti, P., & Arnab, B. D. (2020). Epidemic Modeling and Estimation.

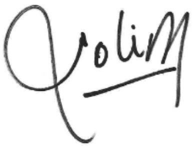

On behalf all authors

Dr. Jorge Soliz

Centre de recherche de l'Institut Universitaire
de Cardiologie et de Pneumologie de Québec
2725 Chemin Ste-Foy,
Québec, QC
G1V 4G5

(418) 656-8711 Poste 8034

Télécopieur: (418) 656-4602
jorge.soliz@crchuq.ulaval.ca
